# Chitosan Molecular Weight Influences on Endodontic Biofilms and Material Enhancement Strategies

**DOI:** 10.3390/dj14040192

**Published:** 2026-03-24

**Authors:** Sumaya Abusrewil, Saeed S. Alqahtani, Mohammed Tiba, Charchit Kumar, Jerina Gjoka, Osama Ramadan, Suror Shaban, Daniel M. Mulvihill, Gordon Ramage, James Alun Scott, William McLean

**Affiliations:** 1 Glasgow Endodontology Group, Glasgow Dental School, School of Medicine, Dentistry and Nursing, College of Medical, Veterinary and Life Sciences, University of Glasgow, 378 Sauchiehall Street, Glasgow G2 3JZ, UK; su.abusrewil@uot.edu.ly (S.A.); mohammad.tiba@glasgow.ac.uk (M.T.); 2225556s@student.gla.ac.uk (S.S.); gordon.ramage@gcu.ac.uk (G.R.); james.scott@glasgow.ac.uk (J.A.S.); 2 Department of Operative Dentistry and Endodontics, School of Dentistry, University of Tripoli, Tripoli 13275, Libya; 3 Department of Restorative Dental Sciences, College of Dentistry, Jouf University, Sakaka 72388, Saudi Arabia; ssalqahtani@ju.edu.sa; 4 Oral Sciences Research Group, Glasgow Dental School, School of Medicine, Dentistry and Nursing, College of Medical, Veterinary and Life Sciences, University of Glasgow, 378 Sauchiehall Street, Glasgow G2 3JZ, UK; 5 Materials and Manufacturing Research Group, James Watt, School of Engineering, University of Glasgow, Glasgow G12 8QQ, UKdaniel.mulvihill@glasgow.ac.uk (D.M.M.)

**Keywords:** endodontic, chitosan, biofilm, antimicrobial, antibiofilm, biodentine

## Abstract

**Objectives:** The identification of novel antimicrobial agents for use in root canal treatment may provide opportunities to improve treatment outcomes. This study aimed to assess the antimicrobial efficacy of different molecular weights of chitosan (CS), and how modification with CS may impact on the antimicrobial, physico-mechanical and biological properties of Biodentine™, a calcium-silicate-based material used in endodontics. **Methods:** *C. albicans* biofilms were treated with either 3% sodium hypochlorite (NaOCl) or a 0.05% or 0.1% CS solution for 5 min. The growth medium was replenished, and cells were re-incubated for additional 72 h. Regrowth of biofilms was assessed using a colorimetric XTT assay. Additionally, multispecies biofilms were established and the regrowth of biofilms on Biodentine discs were quantified following the addition of 0.5 wt% and 1 wt% of CS powder using qPCR. The physico-mechanical and biological properties of the new composite of Biodentine and CS were also evaluated. **Results:** Viability readings revealed significant initial biofilm inhibitory effects of CS solutions, followed by significant regrowth after 72 h. Upon the addition of CS to Biodentine, significant reductions in multispecies biofilm regrowth were determined. Notably, the antibiofilm activity of CS was found to be increased as the molecular weight decreased. The addition of powdered CS of low molecular weight showed a reduction in the mechanical properties of Biodentine, whereas no detrimental effects on the other material properties were noted. **Conclusions:** Chitosan may not be useful as an alternative irrigant to NaOCl. Addition of CS to Biodentine represents a potential means of augmenting the antimicrobial activity of Biodentine against persistent microorganisms following endodontic therapy. Despite the reductions in mechanical properties of the material, the new composite still represents a viable material option when material strength and hardness are not critical.

## 1. Introduction

There has been great interest in identifying new natural compounds that possess antimicrobial properties against endodontic pathogens. Chitosan (CS) is a modified naturally occurring carbohydrate polymer produced by deacetylation of chitin [1], which is widely distributed in nature. Chitin is derived from the exoskeletons of arthropods (including crustaceans and insects), the endoskeleton of cephalopods [2,3], corals [4], sponges [5], algae [6] and the cell walls of fungi [7]. Chitosan, the poly-(β-1,4)-2-amino-2-deoxy-d-glucopyranose, is a collective name for a group of partially and fully deacetylated chitin [8]. Chitosan can be distinguished by its molecular weight into high molecular weight (HMw), medium molecular weight (MMw) and low molecular weight (LMw), and can be easily fabricated into various forms, including membranes [9], solutions/gels [10,11], hydrogels [12], nanofibers [13], films [14], beads [15], nanoparticles [16], scaffolds [17,18] and sponges [19]. Chitosan has attracted considerable attention over the past decade because of its antimicrobial properties [20], biocompatibility, biodegradability and non-toxicity [21,22]. The antimicrobial effectiveness of 0.25% and 0.5% CS, solubilised in acetic acid, has been demonstrated when used as a root canal irrigant [10]. Another study showed that acetic-acid-solubilised CS was effective in reducing the viability of three single-species biofilms, containing *Streptococcus mutans*, *Actinomyces naeslundii*, and *Enterococcus faecalis* [23]. Chitosan is a natural and biocompatible chelating substance. Final irrigation of human root canals with either 15% ethylenediaminetetraacetic acid (EDTA) or 0.2% CS had similar effects on dentine microhardness, push-out strength, sealer penetration into dentinal tubules [24] and smear layer removal capacity [25]. Furthermore, using CS as a final irrigant resulted in significantly greater reductions in biofilm viability than using EDTA. It has been suggested that chitosan nanoparticles (CNPs) can be a useful alternative to EDTA due to its antibiofilm activity and chelating effect [26].

The potential for the use of antimicrobial intracanal medicaments or obturation materials following chemo-mechanical disinfection to inhibit biofilm regrowth is of interest. It has been shown that the sustained release of calcium ions in the root canal system was obtained when CS gel was used as a vehicle for calcium hydroxide [27]. In other studies, the antimicrobial efficacy of root canal sealers was enhanced by the incorporation of CNPs [28,29]. In our previous studies, it has been shown that CS-MMw powder, in its insoluble form, displayed a dose-dependent reduction in multispecies biofilm regrowth when incorporated into Biodentine at 2.5 wt% and 5 wt% [30]. However, the incorporation of 2.5 wt% and 5 wt% CS-MMw compromises many of the material properties of Biodentine, which is likely to reduce its clinical value [31].

Over the past 40 years, many studies have investigated the effect of the molecular weight of chitosan on its antimicrobial properties [32]. However, conflicting findings have been reported [11,33]. Chitosan with HMw forms a dense layer on bacteria that blocks nutrient uptake and waste release, resulting in metabolic disruption and bacterial death [34]. In contrast, chitosan with LMw possesses the capacity to penetrate the cell wall and inhibit DNA/RNA or protein synthesis [35]. While the mechanism of action is beyond the scope of this paper, this study aims to investigate the antimicrobial efficacy of CS with different molecular weights when used as an endodontic irrigant and when incorporated into a calcium silicate cement (Biodentine).

## 2. Materials and Methods

### 2.1. Growing Mono- and Multispecies Biofilms

An established interkingdom endodontic biofilm model containing *Candida albicans* SC5314 (ATCC MYA-2876), *Streptococcus gordonii* (ATCC 35105), *Porphyromonas gingivalis* (ATCC 33277) and *Fusobacterium nucleatum* (ATCC 10953) [36] was used throughout this study. The strain of *C. albicans* was cultured on Sabouraud dextrose agar (SAB) and incubated aerobically at 30 °C for 24–48 h; the strain of *S. gordonii* was grown on Columbia blood agar supplemented with 5% horse blood (CBA) at 37 °C in a 5% CO_2_ incubator for 24 h. The strains of *P. gingivalis* and *F. nucleatum* were cultured on fastidious anaerobic agar (FAA) plates containing 5% defibrinated horse blood and maintained at 37 °C in an anaerobic incubator (Don Whitley Scientific Limited, Bingley, UK) with an atmosphere of 85% N_2_, 10% CO_2_ and 5% H_2_ for 24–48 h. Standardised cultures of *C. albicans* and bacterial species, standardised at 1 × 10^8^ CFU/mL, were first diluted to 1 × 10^6^ CFU/mL and 1 × 10^7^ CFU/mL in the culture broth, respectively. The broth for mono-species *C. albicans* was Roswell Park Memorial Institute-1640 (RPMI), while the broth for multispecies biofilms consisted of 1:1 RPMI with Todd Hewitt Broth (THB) supplemented with 0.01 mg/mL hemin and 2 μg/mL menadione.

To simulate endodontic treatment procedures, biofilm testing involved either direct treatment with CS (termed “treatment”) or mechanical disruption of the biofilm prior to re-growth experiments (termed “prevention”). In the treatment model, mature *C. albicans* biofilms were treated with CS at different concentrations, prior to metabolic assessment. Conversely, in the prevention group, multispecies biofilms were first mechanically disrupted to assess the ability of CS to impede biofilm regrowth on Biodentine discs. All experiments were performed at least three times with at least three technical replicates, unless otherwise stated.

### 2.2. Preparing of Chitosan Solution

A concentration of 10 mg/mL of CS powder of high, medium and low molecular weights (Sigma-Aldrich, St. Louis, MO, USA) was dissolved in 1% acetic acid under steady magnetic stirring for 3 to 4 h at room temperature. The measurement of the pH value of each stock solution was taken using a calibrated pH meter (Mettler Toledo, Leicester, UK) and was determined to be 3.70 (HMw), 3.64 (MMw) and 3.75 (LMw). The stock solution was then diluted down in sterile water to desired concentrations prior to the biofilm treatment.

### 2.3. Determination of the Minimal Inhibitory Concentrations of Chitosan

Minimum inhibitory concentration (MIC) testing was employed to determine the MIC of different Mws of CS against sessile cells of *C. albicans* and co-culture of *C. albicans*, *F. nucleatum*, *P. gingivalis* and *S. gordonii*. Briefly, mono-species (*C. albicans*) inoculum, adjusted to 1 × 10^6^ CFU/mL in RPMI medium, was pipetted in 96-well flat-bottom microtiter plates (Corning Incorporated, Corning, NY, USA) and biofilms were allowed to form for 24 h in an aerobic incubator at 37 °C. For multispecies, standardised cultures of *C. albicans* were first diluted to 1 × 10^6^ CFU/mL, and bacteria (*S. gordonii*, *P. gingivalis* and *F. nucleatum* at 1 × 10^8^ cells/mL) were diluted to 1 × 10^7^ cells/mL in the culture broth (1:1 RPMI/THB). Biofilms were grown for 24 h at 37 °C in a 5% CO_2_ incubator. Following the incubation time, the supernatant was discarded, and the biofilms were washed with phosphate buffered saline (PBS). The biofilms were treated with 2-fold serial dilutions of CS-HMw, CS-MMw and CS-LMw solutions with concentrations starting from 2.5 mg/mL. The plates were then re-incubated as described above. After 24 h, the biofilms were gently washed with PBS prior to the addition of 100 µL XTT (2,3-bis-[2-methoxy-4-nitro-5-sulfophenyl]-2H-tetrazolium-5-carboxanilide [Sigma-Aldrich, Dorset, UK]) to assess the biofilm viability. After 2 h of incubation at 37 °C, 75 µL XTT were transferred to a new microtiter plate for measurement spectrophotometrically using the microtiter plate reader (Sunrise, TECAN, Theale, UK) at a wavelength of 492 nm. The sMIC_50_ and sMIC_90_ were considered the concentrations that led to 50% and 90% reductions in XTT readings, respectively, when compared with the positive control. For all experiments, positive controls (untreated biofilms) were run in parallel. Appropriate negative controls minus inoculum were also included to assess for media contamination.

### 2.4. Biofilm Treatment and Regrowth Assessment

Next, treatments were tested against *C. albicans* mono-species biofilms grown in 24-well flat-bottom microtiter plates (Costar^®^, Corning Incorporated, Corning, NY, USA) for 24 h, as previously described. Following incubation, unattached cells were removed by washing the resulting biofilms with PBS. Biofilms were treated for 5 min by adding 500 µL of either 0.05% or 0.1% of CS-HMw, CS-MMw, or CS-LMw, or 3% sodium hypochlorite (NaOCl). Afterwards, treatments were removed, and each well was washed once with PBS. To further assess the regrowth ability of biofilms, 500 µL of a fresh RPMI medium was added to the treated and washed biofilms. Plates were re-incubated for a further 72 h. Appropriate positive and negative controls were also included in each plate. The viability of cells remaining immediately after treatment (0 h) and those regrowing after 72 h of re-incubation was assessed using the XTT reduction assay, as previously described [37].

### 2.5. Effects of Chitosan on Antimicrobial Properties of Biodentine

#### 2.5.1. Preparation of Biodentine Materials ± Chitosan

The effect of 0.5 wt% and 1 wt% of high, medium and low molecular weights of CS powder on the antimicrobial efficacy of Biodentine were assessed against the regrowth of 4-species biofilms (Table 1). Biodentine powder in each capsule was reweighed to an exact 0.7 g and then mixed with two different proportions of CS powder: 0.5 wt% and 1 wt% (Table 2). The resultant powder was mixed with the manufacturer’s Biodentine™ liquid component in a mixing machine at 4000–4200 rpm for 30 s and then compacted into moulds (7 mm in diameter × 1 mm in thickness), as previously shown [30]. All specimens were kept at 37 °C in a humid atmosphere (around 95–100% relative humidity) for 1 h. Material discs were then disinfected using the UV for 15 min.

#### 2.5.2. Quantitative Analysis of Mixed-Species Biofilms Formed on Biodentine ± Chitosan

The antimicrobial ability of Biodentine incorporated with CS was assessed against biofilm regrowth on the material placed in a 24-well plate, as previously described [30]. Briefly, the four-species biofilms were grown in RPMI/THB for 24 h in a 24-well plate. The spent media was then discarded, and the biofilm in each well was washed once with PBS and mechanically disrupted in 1 mL of fresh RPMI/THB media to simulate the mechanical debridement of the root canal. The disrupted biofilms were diluted to 1:10 in RPMI/THB and then inoculated on the material discs into a new 24-well plate. The plate was then re-incubated for an additional 24 h in 5% CO_2_ at 37 °C to allow for biofilm growth. Following incubation and discarding spent media, each disc was washed with PBS, transferred into a bijoux tube containing 1 mL PBS and then sonicated at 35 kHz in a sonic bath for 10 min. The sonicate was transferred to 1.5 mL Eppendorf tubes (Greiner Bio-one, Kremsmünster, Austria, UK) for DNA extraction.

The composition of the regrown biofilms on Biodentine discs was assessed using quantitative polymerisation chain reaction (qPCR). Live/dead qPCR is a technique that uses a DNA-intercalating dye propidium monoazide (PMA) to differentiate between viable and total cells. Samples were prepared as previously described by Sherry and colleagues [38]. Briefly, prior to DNA extraction, each sonicated sample was equally split and 5 µL/mL of 50 µM PMA dye was added to one half of each sample. Following this, treated and control samples (without PMA) were all incubated in the dark at room temperature for 10 min to allow treated cells to uptake the dye. Samples were placed on a bed of ice and then exposed to a 650 W halogen light, positioned 20 cm away from the sample tubes, for 5 min. Following this, DNA extraction and real-time quantitative analysis were carried out. DNA was extracted from samples according to the manufacturer’s instructions using the QIAamp DNeasy Mini Kit (Qiagen, Manchester, UK). The composition of the regrown biofilms was enumerated using real-time qPCR. The mastermix contained SYBR™ GreenER™ and UV-treated RNase-free water with forward and reverse primers for either the bacterial or *Candida* species, as listed in Table 3. Quantitative PCR was performed using the StepOnePlus™Real-Time PCR system (Applied Biosystems, Waltham, MA, USA), and data analysed using StepOnePlus software version 2.3 (ThermoFisher, Paisley, UK). All samples were run in duplicate in the qPCR with negative controls (mastermix only) to assess for DNA contamination. The colony-forming equivalent (CFE) of samples was calculated using a previously established standard curve methodology [39] of serially extracted DNA bacterial and fungal colony-forming units from 1 × 10^4^ to 10^8^ CFU/mL.

### 2.6. Material Characteristics of Biodentine ± LMw Chitosan Powder

Modified specification tests of the International Organisation for Standardisation (ISO) 6876 [40] were adapted for material tests, as previously described [31], unless otherwise stated. All specimens were kept at 37 °C in a humid atmosphere (around 95–100% relative humidity) for the specified time.

#### 2.6.1. Setting Time

Biodentine samples (n = 5) were mixed and compacted into stainless-steel ring moulds (13 mm in diameter × 2 mm in height) on a glass slab. The excess material was removed to obtain a flat surface, and the assembly was stored in the cabinet in a moist atmosphere at 37 °C. Ten minutes after mixing, the assembly was taken out of the incubator. A Vicat apparatus E055N (Matest, Caerphilly, UK), consisting of a sliding rod and a removable needle, was used to perform the test. The needle of the apparatus was carefully lowered onto the cement surface. The final setting time was calculated as the time taken from the end of mixing to the time at which a mark was no longer visible on the set material surface.

#### 2.6.2. Solubility

The solubility test determined the weight loss of the test samples. Samples (n = 6) were prepared using ring metal moulds (13 mm × 2 mm). All moulds were individually weighted before use (W_0_). Each mould was filled with the allocated material group on a glass slab, and excess material was removed. The test specimens were left to set in a humid atmosphere at 37 °C for 24 h. All specimens were then weighed individually in their moulds (W_1_) before immersion in water. The difference found between W_1_ and W_0_ was recorded as the initial dry weight (IDW). Each sample was immersed in a separate glass jar filled with 20 mL of sterile distilled water. The small jars were weighed separately before being utilised (W_2_). Autoclave adhesive strips were attached to each ring mould to suspend a sample in each glass jar, allowing both surfaces of the cement to be freely accessible by the immersing water without touching the container walls. The glass jars containing cement samples were placed in the incubator for one day. After 24 h, each specimen was taken out and rinsed with 1 mL of distilled water recollected in the same glass container to remove loose debris. The tape was then removed from each mould, and the specimens were left to dry for 48 h in the incubator before being reweighed with their ring moulds (W_3_). The difference found between W_3_ and W_0_ was recorded as the final wet weight (FWW). The water inside jars was then evaporated in an oven at 95 °C, and the dry glass jars with residues inside were weighed once cooled (W_4_). The difference found between (W_4_) and the initial weight of the glass container (W_2_) was recorded as the dry precipitant weight (DPW). All measurement readings were in grams and recorded to four decimal places to the nearest 0.0001 g. The amount of solubility was calculated to the nearest 0.001% using the following equations:

$$\mathrm{Solubility}\;A\;(\%)\;=\;\frac{\mathrm{DPW}}{\mathrm{IDW}}\;\times \;100\;(\mathrm{Residue}\;\mathrm{method})$$

$$\mathrm{Solubility}\;B\;(\%)\;=\;\frac{\mathrm{IDW}\;-\;\mathrm{FWW}}{\mathrm{IDW}}\;\times \;100$$

#### 2.6.3. Radiopacity

Cement specimens (n = 5) were prepared (7 mm in diameter and 1 mm in thickness). Test samples were kept in a humid incubator at 37 °C for 24 h. Each test sample was digitally radiographed alongside an aluminium step wedge (5 steps with a thickness of 2.5, 3.5, 4.75, 7 and 9 mm). The digital X-ray machine used (Gendex 765DC, Gendex, Des Plaines, IL, USA) operated at 65 kV with a current of 7 mA and an exposure time of 0.020 s. The radiographic images were exported in JPG format (Supplementary Figure S1). The radiopacity value of each specimen was determined using ImageJ software version 1.53t (National Institutes of Health, Bethesda, MD, USA). The mean grey pixel values of various thicknesses of the aluminium stepwedge were obtained and plotted against their thickness in mm. A calibration curve of grey values versus thickness of aluminium was constructed automatically. Following calibration, the mean grey value for each specimen was then automatically expressed in mm of aluminium (mmAl).

#### 2.6.4. Compressive Strength

A modified test of ISO 9917-1 [41] was adapted for testing the compressive strength (CSI). Cylindrical specimens (n = 6) were prepared (6 mm in height × 6 mm in diameter) using silicone moulds. All samples were stored in a humid atmosphere for 30 days at 37 °C. Prior to testing, each material test was positioned back into a mould. Both circular faces of each cylindrical specimen were polished using a stainless-steel dental polishing strip to ensure the creation of two smooth and flat surfaces. An Instron 3367 Universal Testing Machine [30 kN Static Load Cell (Instron, Buckinghamshire, UK)] was used to measure the CSI for each material cylinder at a speed of 1 mm/minute. The load was applied in a direction parallel to the long axis of each cylinder until the specimen was crushed. The maximum load required to break each test sample was recorded. The compressive strength was calculated in megapascals using the formula σc (MPa) = 4F/πd^2^ (N/mm^2^), where F is the maximum force applied and d is the mean diameter of the specimen in mm.

#### 2.6.5. Microhardness

A modified test of ISO 6507-1 [42] was adapted for the Vickers microhardness (HV) test. Disc-shaped specimens (n = 5) were prepared by compacting the Biodentine material into stainless-steel ring moulds (13 mm × 2 mm) 30 days before the test. The testing was performed using a Vickers microhardness tester (Krautkramer TIV, [GE Inspection Technologies, Coventry, UK]) with a Vicker diamond indenter point. Three test indentations were performed on the polished surface of each specimen. The image of each indentation was automatically transferred and evaluated. The mean Vickers hardness number (VHN) was recorded for each test group.

#### 2.6.6. Cytotoxicity

Human dental pulp stem cells (h-DPSCs) [43] supplied by Lonza (Lonza Inc., Bornem, Belgium) were cultured. Briefly, the cells were seeded in 75 cm^2^ flasks at 5000–6000 cells/cm^2^ in Knock-out Dulbecco’s modified Eagle’s medium (DMEM-KO [Gibco, Loughborough, UK]) supplemented with 10% foetal bovine serum, 200 mM L-Glutamine and Penicillin–Streptomycin solution. The medium was refreshed every three days, and when flasks were approximately 90% confluent, cells were passaged by detaching with 0.25% trypsin–EDTA (Gibco™, Loughborough, UK). Biodentine ± CS-LMw discs (7 mm in diameter × 2 mm in thickness) were prepared and left in a humid atmosphere for 24 h. Discs were then disinfected by UV for 30 min. Material discs were placed in a 24-well plate submerged in 1 mL of DMEM-KO and maintained at 37 °C for 24 h prior to testing. At the same time, stem cells were plated into a 96-well plate in DMEM-KO at a density of 1 × 10^4^ cells/well. The following day, the cell’s medium was replaced with the material leachate and incubated for 72 h. The cytotoxicity of CS-LMw was assessed by measuring the release of lactate dehydrogenase (LDH), a marker of membrane damage and cell death. An LDH cytotoxicity assay kit (CyQUANT™ LDH Cytotoxicity Assay [Thermo Scientific, Loughborough, UK]) was used according to manufacturer’s instructions. Briefly, 50 μL of cell supernatants were plated in a 96-well plate with a positive control (lysed cells). Then, 50 μL of LDH reaction mix was added to each well and incubated in the dark for 30 min at room temperature. After incubation, the reaction was terminated by adding 50 μL of a stop solution provided by the manufacturer. Absorbance was read at 490 nm with a reference wavelength of 680 nm using a microplate reader (FluoStar Omega, BMG Labtech, Aylesbury, UK).

#### 2.6.7. Statistical Analysis

Graphs, data distribution, and statistical analysis were performed with GraphPad Prism version 9 (GraphPad, San Diego, CA, USA). Before the analysis, the D’Agostino–Pearson omnibus and Shapiro–Wilk normality tests were used to assess data distributions. The Kruskal–Wallis and Dunn’s tests were used to determine the *p* values for multiple comparisons of non-parametric data. The ANOVA with Tukey’s tests were used for multiple comparisons of normally distributed parametric data. Differences were considered statistically significant when *p* < 0.05. Each error bar represents the standard deviation (SD).

## 3. Results

### 3.1. Determination of the sMICs of Chitosan for Sessile Cells

**Figure 1:**
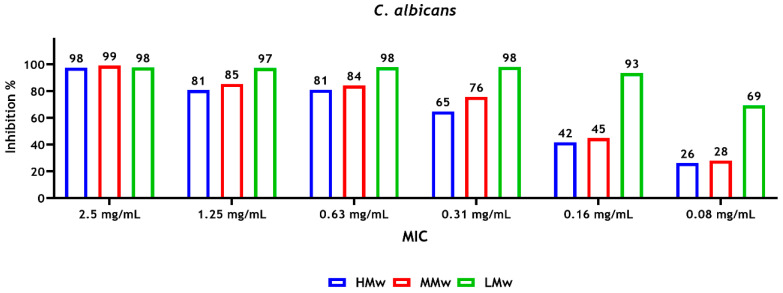
Susceptibility of *C. albicans* sessile cells after treatment with different molecular weights of CS. The absorbance values were read at 492 nm. Inhibition values, compared with the positive control (untreated biofilms), represent the mean of data obtained from triplicates of three independent experiments.

The results of susceptibility of microbial strains to CS revealed that a concentration of 0.16 mg/mL of CS-LMw was required to inhibit approximately 90% of the *C. albicans* biofilm metabolic activity (sMIC_90_), whereas a higher concentration of CS-HMw and CS-MMw (0.63 mg/mL) was required to inhibit approximately 80% of the metabolic activity of the biofilm (Figure 1).

**Figure 2:**
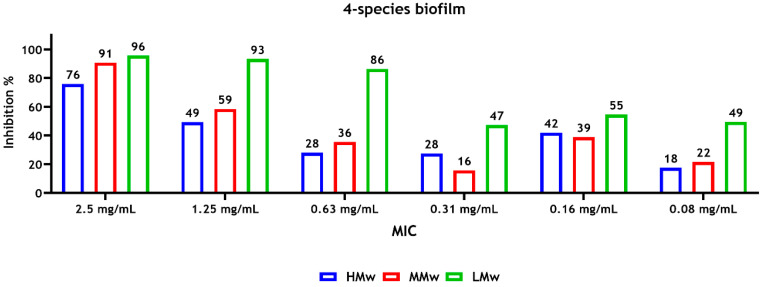
Susceptibility of 4-species sessile cells (*F. nucleatum*, *P. gingivalis*, *S. gordonii* and *C. albicans*) after treatment with different molecular weights of CS. Inhibition values, compared with the positive control (untreated biofilms), represent the mean of data (average) obtained from triplicates of three independent experiments. The absorbance was measured at a wavelength of 492 nm.

For the multispecies biofilms, a concentration of 1.25 mg/mL of CS-LMw and CS-HMw inhibited about 90% (sMIC_90_) and 50% (sMIC_50_) of the metabolic activity of the biofilms, respectively (Figure 2). In general, the highest and lowest reductions in the metabolic activity were observed with CS-LMw and CS-HMw, respectively (Figure 1 and Figure 2).

### 3.2. Biofilm Treatment with Sodium Hypochlorite or Chitosan and Regrowth Assessment

**Figure 3:**
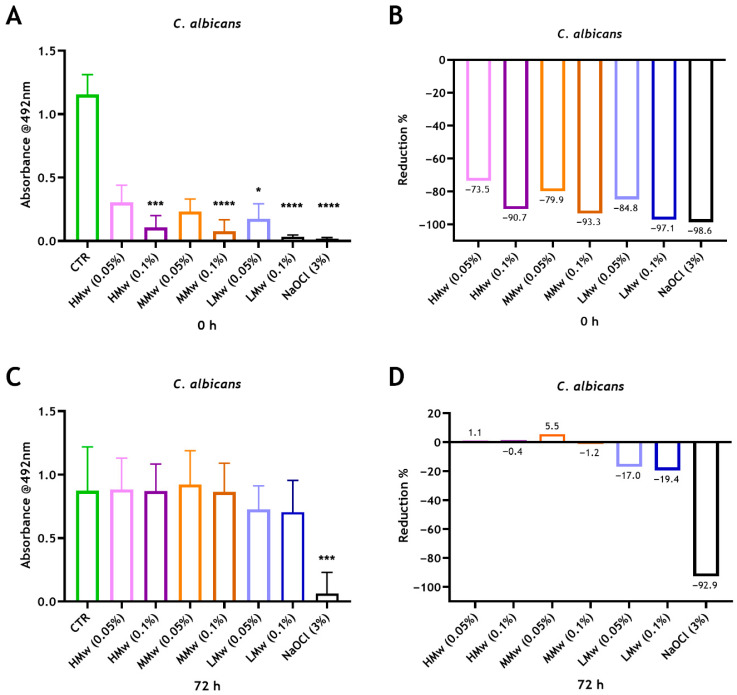
Assessing the metabolic activity of performed 24 h *C. albicans* biofilms following treatment with NaOCl and two different concentrations of CS with HMw, MMw and LMw. *C. albicans* biofilms were grown for 24 h and then treated for 5 min with either 3% NaOCl or with 0.5 mg/mL (0.05%) or 1 mg/mL (0.1%) of different molecular weights of CS. The readings of XTT were taken at 0 h (**A**) and 72 h (**C**) after re-incubation of biofilms with fresh RPMI. Values were plotted as biofilm reduction percentages at 0 h (**B**) and 72 h (**D**) in relation to the untreated controls. Statistically significant differences between the control and each treatment conditions were presented as * *p* < 0.05, *** *p* < 0.001 and **** *p* < 0.0001. Each bar represents the mean of data obtained from four technical repeats of three independent experiments.

The NaOCl or CS treatment effectiveness against *C. albicans* mono-species biofilms were assessed. It was determined that 3% NaOCl had an antifungal effect at 0 h and 72 h, resulting in significant reductions in metabolic activity (~99% and ~93%) compared with the untreated control (**** *p* < 0.0001 and *** *p* < 0.001), respectively (Figure 3). It has also been revealed that 0.05% CS-HMw, CS-MMw and CS-LMw exhibited approximately 74%, 80% and 85% reductions in viability, while treating the biofilms with 0.1% CS-HMw, CS-MMw and CS-LMw exhibited significant immediate reductions (91%, 93% and 97%, respectively) following treatment. Thus, CS solutions display concentration-dependent reductions in biofilm viability where the antifungal effect increases with the decreased molecular weight. Despite the initial antifungal effect of CS solutions, the cells remaining were able to regrow over time. Only slight reductions were observed with the 0.05% and 0.1% CS-LMw groups at 72 h compared with the positive control (Figure 3).

### 3.3. Low-Molecular-Weight Chitosan Confers the Greatest Antimicrobial Properties on Biodentine

**Figure 4:**
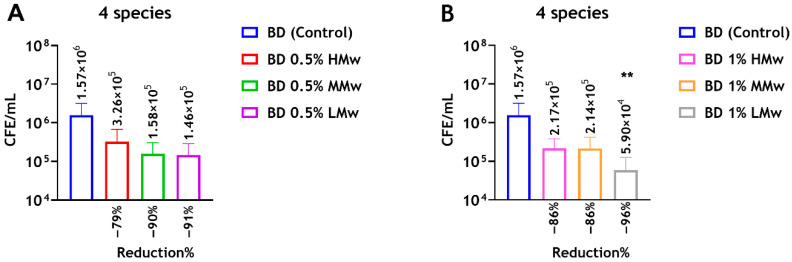
Compositional analysis of regrown biofilms on Biodentine incorporated with different molecular weights of CS. Chitosan amounts of 0.5 wt% and 1 wt% of HMw, MMw and LMw were incorporated into the Biodentine material, and live/dead qPCR was performed. (**A**) Live CFE/mL of mixed-species (4-species) biofilms grown on Biodentine ± 0.5 wt% CS. (**B**) Live CFE/mL of 4-species biofilms grown on Biodentine ± 1 wt% CS. ** indicates statistically significant differences between the control and the test group (** *p* < 0.01). Reduction (%) of CFE/mL for each test group compared with the control unmodified cement are shown. Data are representative of biofilms from three repeats with three technical replicates.

The quantitative analysis revealed decreases in the live CFE counts of 4-species biofilms following incorporation of CS powder into Biodentine compared with the unmodified Biodentine (control). At 0.5 wt% CS, reductions in CFE counts of approximately 79%, 90% and 91% were observed with HMw, MMw and LMw, respectively. Similarly, the live CFE/mL following the addition of 1 wt% CS-HMw, CS-MMw and CS-LMw was reduced by approximately 86% and 86% and 96%, respectively. Notably, the addition of 1 wt% CS-LMw reduced the CFE counts significantly (** *p* < 0.01) from 1.57 × 10^6^ to 5.9 × 10^4^ CFE/mL. These data suggest that CS-LMw showing the greatest antibiofilm potential when compared with MMw and HMw (Figure 4).

### 3.4. Material Characteristics of Biodentine

Based on the previous results, where CS-LMw demonstrated the greatest antimicrobial properties, this was chosen for future experiments to explore the impact of CS on the material properties of Biodentine.

#### 3.4.1. Setting Time

**Figure 5:**
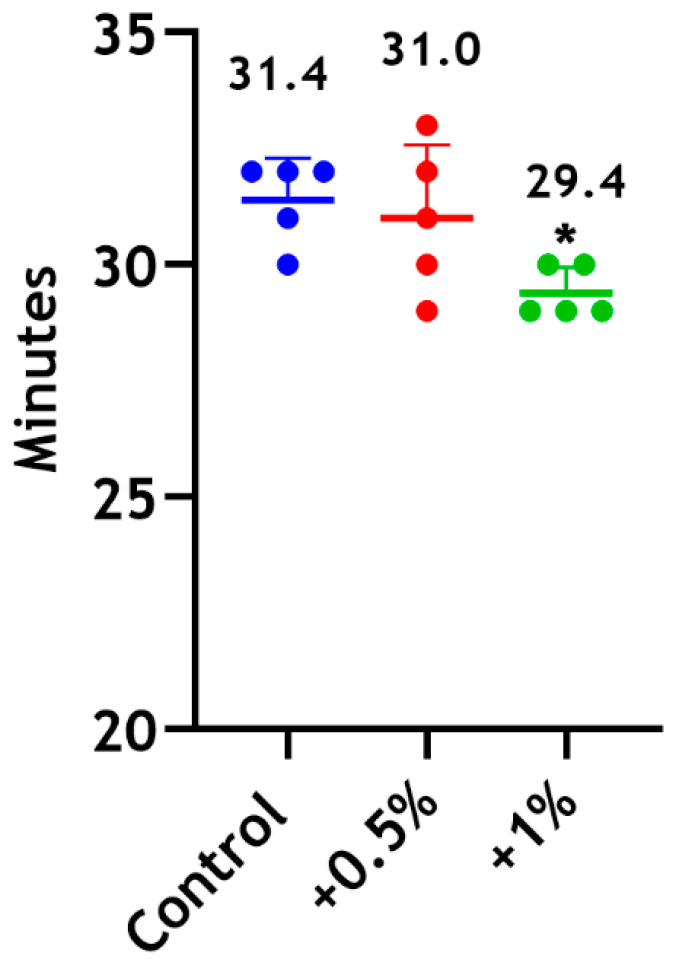
Setting time of Biodentine following the incorporation of 0.5 wt% (red) and 1 wt% (green) of CS-LMw. * indicates statistically significant differences between the 1 wt% group and the control (* *p* < 0.05). The control unmodified material is shown in blue. Each dot represents a replicate (n = 5).

The setting times for the unmodified material and modified Biodentine supplemented with 0.5 wt% CS-LMw were comparable. No statistically significant differences were found between the 0.5 wt% and 1 wt% groups (Figure 5).

#### 3.4.2. Radiopacity

**Figure 6:**
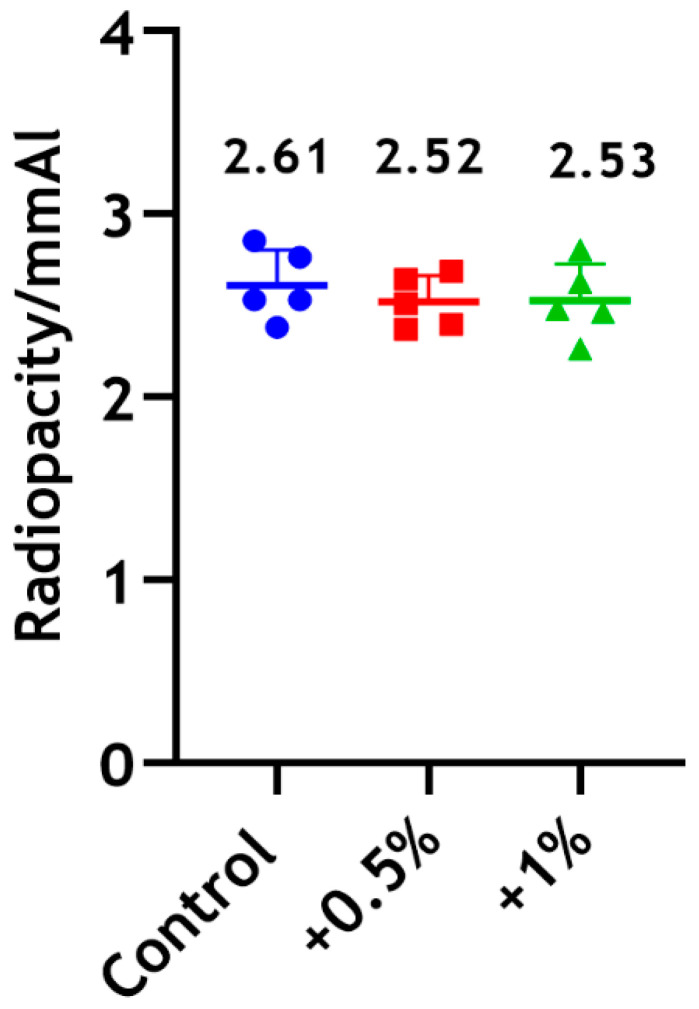
Radiopacity assessment of Biodentine following the incorporation of 0.5 wt% (red) and 1 wt% (green) of CS-LMw. The control unmodified group is shown in blue. Each dot/shape represents a replicate (n = 5).

The radiopacity of Biodentine incorporated with CS-LMw demonstrated a negligible decrease by approximately 3%, with no statistically significant differences between the test groups (Figure 6).

#### 3.4.3. Solubility

**Figure 7:**
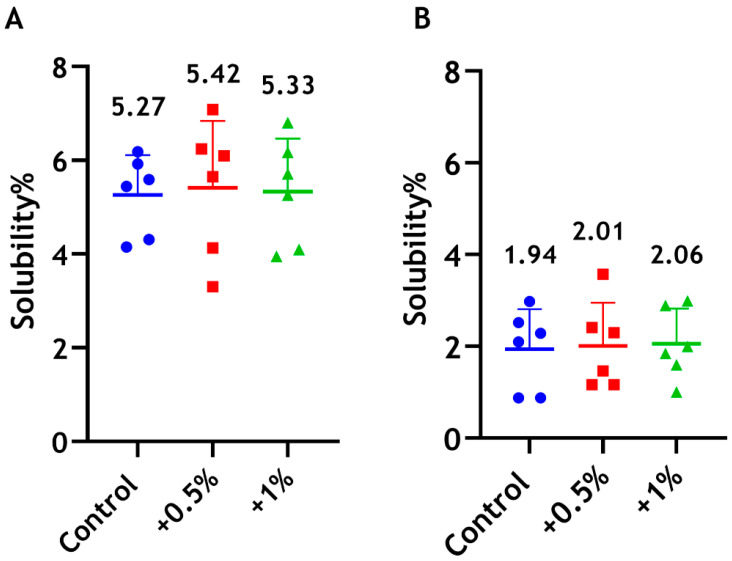
Solubility percentages of Biodentine when incorporated with 0.5 wt% (red) and 1 wt% (green) of CS-LMw, following immersion in sterile water for 24 h. The control unmodified group is shown in blue. (**A**) The amount of Biodentine removed from the specimens (residue method) was calculated as a percentage of the original weight. (**B**) The difference in Biodentine weight before and after immersion was calculated as a percentage of the original weight. Each dot/shape represents a replicate (n = 6).

The results showing that the solubility/disintegration of the unmodified material and modified Biodentine incorporated with CS were comparable with no significant differences (Figure 7).

#### 3.4.4. Cytotoxicity

**Figure 8:**
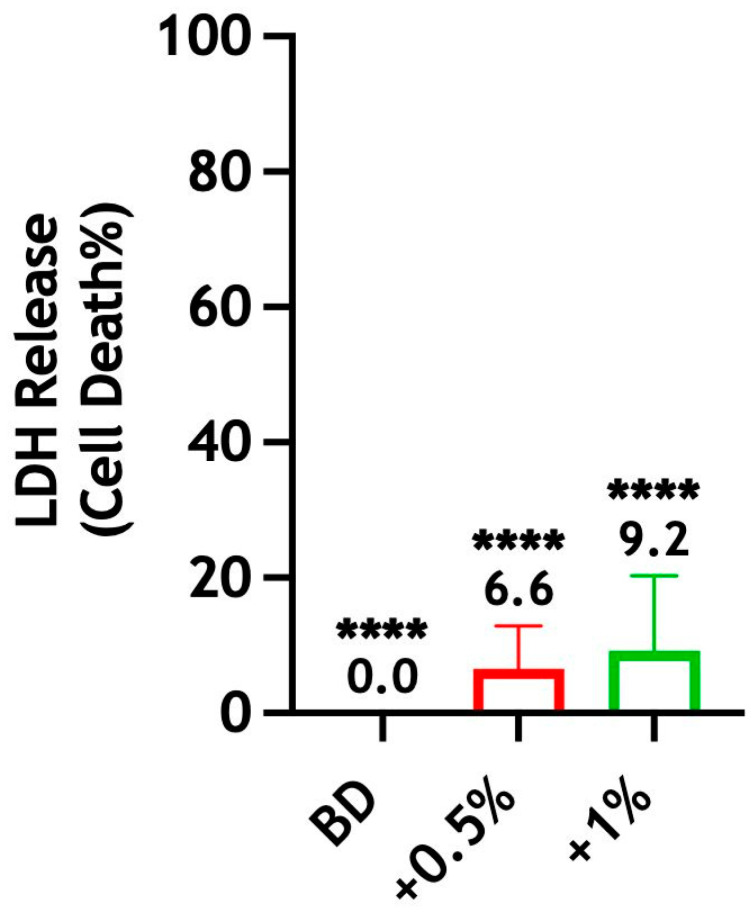
Evaluation of the cytotoxic effect of Biodentine on h-DPSCs when incorporated with 0.5 wt% (red) and 1 wt% (green) of CS-LMw. The percentage (%) of LDH release was measured from h-DPSCs treated with 100% extract of Biodentine ± CS-LMw. The percentage of cytotoxicity of test groups was calculated relative to the control (lysed cells). **** indicates statistically significant differences between lysed cells and test groups (**** *p* < 0.0001). Note: Statistical analyses were performed on raw data. Each bar represents the mean of data obtained from two technical repeats of three independent experiments.

The results indicate that Biodentine ± CS-LMw presented no cytotoxic effect in undiluted extracts on human stem cells at 72 h. The LDH released from the treated h-DPSCs was significantly lower when compared with the maximum release of the LDH achieved by the lysis solution (**** *p* < 0.0001). No statistically significant differences were observed between the test groups (Figure 8).

#### 3.4.5. Microhardness

**Figure 9:**
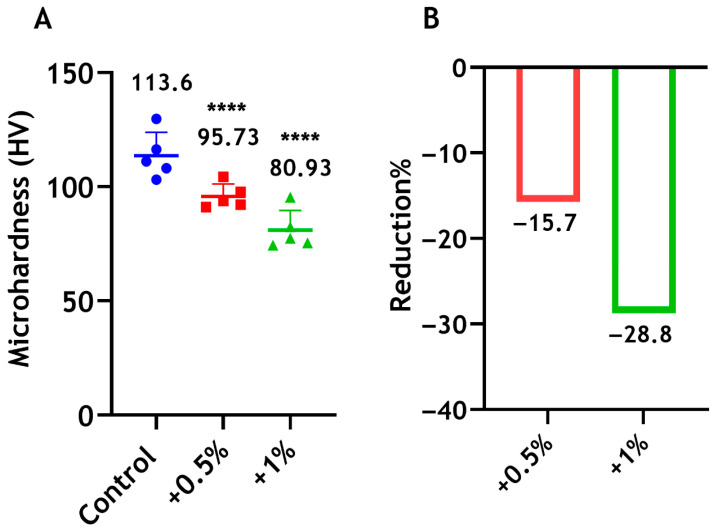
(**A**) Microhardness of Biodentine when mixed with 0.5 wt% (red) and 1 wt% (green) of CS-LMw. **** indicates statistically significant differences between the test groups and the control (**** *p* < 0.0001). The control unmodified cement (blue). Each dot/shape represents a replicate (n = 5). (**B**) The percentages of reduction in microhardness compared with the control.

The microhardness of Biodentine declined significantly by 16% and 29% following the incorporation of 0.5 wt% and 1 wt% CS-LMw, respectively, compared with the control unmodified cement (Figure 9). A significant difference was also observed between the 0.5% and 1% groups (*p* < 0.05).

#### 3.4.6. Compressive Strength

**Figure 10:**
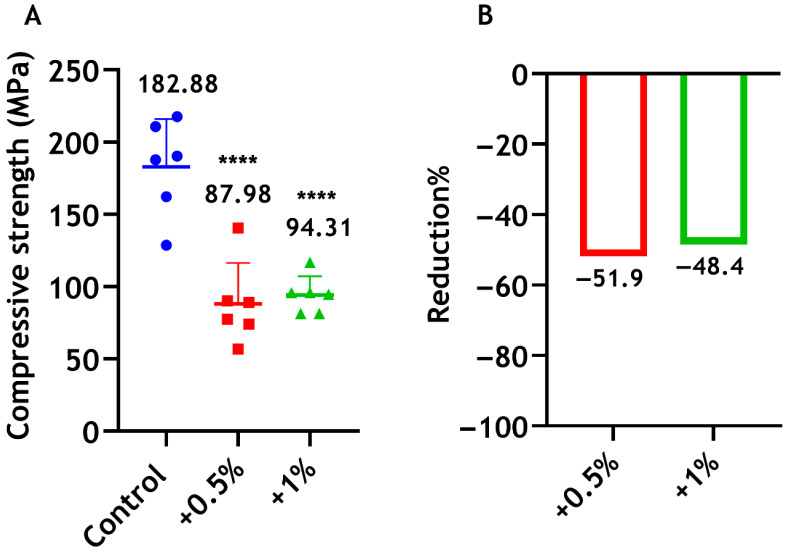
(**A**) Compressive strength of Biodentine when mixed with 0.5 wt% (red) and 1 wt% (green) of CS-LMw. **** indicates statistically significant differences between the chitosan groups and the control (**** *p* < 0.0001). The control unmodified cement is shown in blue. Each dot/shape represents a replicate (n = 6). (**B**) The percentages of reductions in compressive strength expressed in relation to the control commercial cement.

The compressive strength of Biodentine decreased significantly when CS-LMw was integrated into the cement compared with the unmodified formulation, which showed greater strength (182.88 MPa). No statistically significant difference was found between the 0.5% and 1% groups (Figure 10).

## 4. Discussion

It is clear from our findings that CS showed antibiofilm activity in both solution and powder forms. In previous studies, a correlation between antimicrobial activity and chitosan Mw has been reported. It was shown that decreasing chitosan Mw led to strengthening chitosan antimicrobial activity [16,44]. Conversely, in other studies, the antimicrobial effect was enhanced as the molecular weight of CS increased [11,45].

The data of our study suggested that CS with LMw showed the highest antimicrobial effect, and the antibiofilm effect was decreased with increasing chitosan Mw. This could be due to size effects [44], as the molecular particle size decreases with a decrease in the molecular weight. The antimicrobial mechanism of action of CS is not entirely defined, but different mechanisms have been proposed [20,46], and it is assumed to be mainly electrostatic. A previous study showed that CS binds to the negatively charged phospholipids that alter the fluidity of the cell membrane, leading to membrane permeability [47]. Additionally, it has been hypothesised that CS exhibits a chelating activity against essential trace metals and thereby inhibits fungal growth and toxin production [48]. An RNA sequencing analysis has shown that CS may exert its antifungal effect by inhibiting genes involved in protein biosynthesis and cell integrity [49]. In another study, CS was found to inhibit bacterial growth via downregulation of genes involved in growth and metabolism [50].

Due to chitosan’s limited solubility, acetic acid is commonly used to solubilise chitosan. The findings from a previous study indicate that 1% acetic acid possessed antimicrobial activity, and the combination of CS and acetic acid produced a synergetic antimicrobial effect [51]. In our study, however, the stock concentration of CS (10 mg/mL solubilised in 1% acetic acid) was diluted down in sterile water to 0.5 mg/mL (0.05%) and 1 mg/mL (0.1%), diminishing the antimicrobial effect of acetic acid.

We have shown that CS solutions were initially effective against *C. albicans* biofilms, irrespective of Mw, where the greatest significant reduction was observed for 0.1% CS-LMw, which was comparable with NaOCl. Nevertheless, this short treatment with CS solutions was not similarly effective at preventing biofilm regrowth after 72 h of re-incubation on microtiter plates, which was far less than that of the gold-standard irrigant (NaOCl). It has been reported that the exposure time of chitosan plays a significant role in its antibiofilm effectiveness [52]. Therefore, longer exposure to CS may be required to increase its antibiofilm effects. When exploring the effect of powdered undissolved CS on the antimicrobial properties of Biodentine, a significant improvement was noted, particularly with LMw.

Based on the previous results, CS-LMw was chosen for the next material experiments.

Our results show that the addition of small proportions of CS-LMw (0.5 wt% and 1 wt%) had no detrimental effects on the setting time, radiopacity and solubility of the material. The LDH assay was used to determine the cytotoxicity against h-DPSCs. Damage to the plasma membrane rapidly releases LDH into the cell culture supernatant, which is a reliable indicator of cytotoxicity [53]. Our results indicate that the undiluted extracts of Biodentine with small concentrations of CS-LMw showed no cytotoxic effect on h-DPSCs. Importantly, incorporation of 0.5 wt% and 1 wt% CS-LMw into Biodentine reduces the mechanical properties of the material. The addition of CS powder appeared to disturb the connectivity of the material structure and hydration reactions, leading to significant declines in mechanical strength [31]. Despite these reductions, such a new composite could still be utilised in various endodontic applications. In fact, compressive strength and microhardness are not important factors to consider when a filling material does not bear direct pressure nor scratching. Thus, the clinical use of the modified Biodentine with smaller amount of CS could be limited to specific applications where the material bioactivity can be beneficial, but high compressive strength is not required [31]. Of note, even with the addition of CS-LMw, the compressive strength of the modified Biodentine material (approximately 90 MPa—Figure 10) was superior to ProRoot Mineral Trioxide Aggregate (MTA) (38.21 MPa; Supplementary Figure S2). Thus, this new composite could still be applicable for use in any situation where MTA might be considered.

In this work, we used insoluble CS from an animal origin. It may be of interest to explore the usefulness of water-soluble CS that could be dissolved easily in the manufacturer’s liquid of Biodentine. Using fungal chitosan, which has received less research interest, could also warrant investigation.

## 5. Conclusions

The present study highlights that chitosan may not be useful as an alternative irrigant to NaOCl. Our study also highlights the potential to enhance the antimicrobial properties of Biodentine and other calcium silicate materials, which may reduce the likelihood of endodontic re-infections and treatment failures.

## Figures and Tables

**Table 1:** Chitosan with high, medium and low molecular weights used in this study (Sigma Aldrich).

Description	Biological Source	Molecular Weight	DDA
CS-LMw	Animal origin	50,000–190,000 Da	75–85%
CS-MMw	Animal origin	190,000–310,000 Da	75–85%
CS-HMw	Animal origin	310,000–375,000 Da	>75%

DDA; degree of deacetylation.

**Table 2:** Chitosan percentages (%) incorporated into Biodentine material.

Test Group	BD-P (g)	* CS-P (g)	BD-L (μL)
BD control	0.7	—	180
BD + 0.5 wt%	0.6965	0.0035	180
BD + 1 wt%	0.693	0.007	180

Powder weighing 0.7 g of the unmodified Biodentine™ (BD-P) was mixed with 180 μL of the manufacturer Biodentine liquid (BD-L [5 drops of Biodentine liquid = 180 μL]). Positive control: unmodified commercial Biodentine material. * The chitosan powder (CS-P) was not solubilised in acetic acid to avoid replacement of the manufacturer’s Biodentine liquid with the solubilised form of CS in the diluted acetic acid.

**Table 3:** Primer sequences used for compositional analysis of multispecies biofilm model [30].

Organism	Primer	Forward Primer 5′-3′	Reverse Primer 5′-3′
Fungi	18S	CTCGTAGTTGAACCTTGGGC	GGCCTGCTTTGAACACTCTA
Bacteria	16S	TCCTACGGGAGGCAGCAGT	GGACTACCAGGGTATCTAATCCTGTT

## Data Availability

The original contributions presented in this study are included in the article/Supplementary Material. Further inquiries can be directed to the corresponding author.

## Supplementary Materials

The following supporting information can be downloaded at: https://www.mdpi.com/article/10.3390/dj14040192/s1, Figure S1: Digital radiographic images of Biodentine discs; Figure S2: Compressive strength testing of MTA using Instron Testing Machine.
