# Supplementary material for: Chitosan Molecular Weight Influences on Endodontic Biofilms and Material Enhancement Strategies
Source: Dent J (Basel). 2026 Mar 24;14(4):192. doi: 10.3390/dj14040192 (PMC13114319; doi:10.3390/dj14040192)
Supplement: Supplementary file 1 [file dentistry-14-00192-s001.zip › dentistry-4142309-supplementary.pdf]

Supplementary Materials

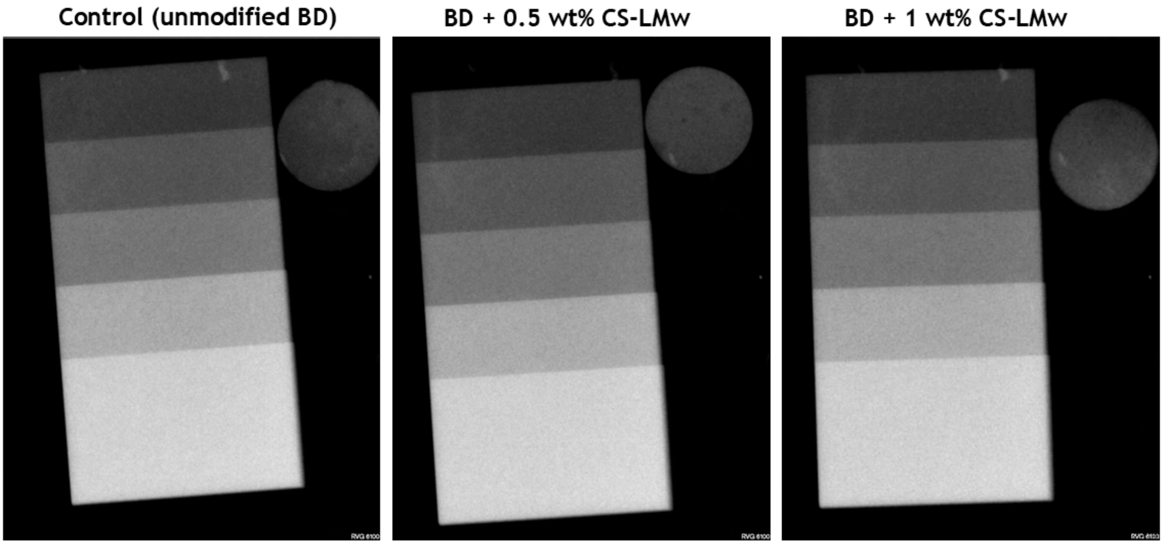

Figure S1: Digital radiographic images of Biodentine discs. Each replicate was digitally radiographed with the aluminium step wedge. The material mean grey value was expressed in mm of aluminium thickness. A representative image for each group is shown.

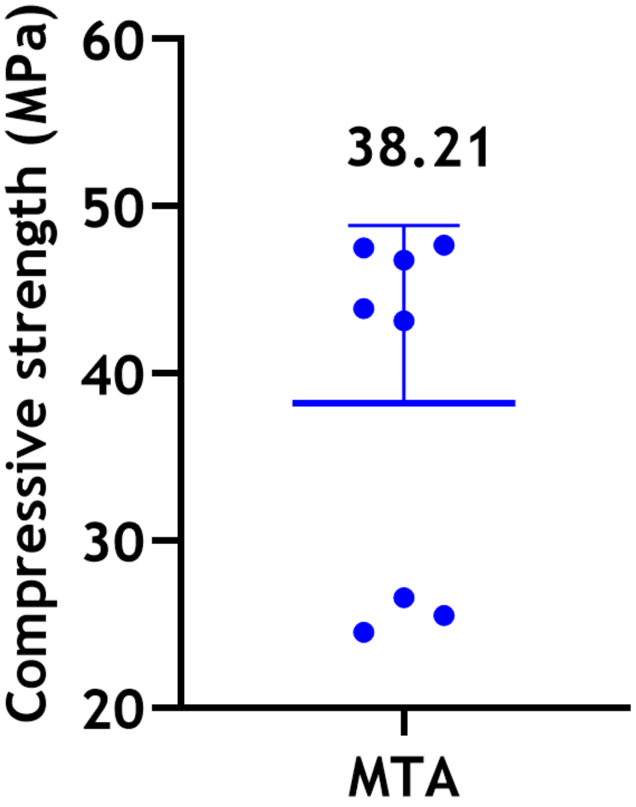

Figure S2: Compressive strength testing of MTA using Instron Testing Machine. Measurements were carried out on 30-day-old cylinders (6 × 6 mm) stored in a humid atmosphere at 37°C. Each dot represents a replicate (n = 8). The error bar represents SD (±10.63 MPa).
